# Post-Traumatic Growth in University Students After Earthquakes: The Effect of Perceived Social Support and Psychological Resilience

**DOI:** 10.3390/bs15091178

**Published:** 2025-08-29

**Authors:** Ferhat Toper, Rauf Yanardağ, Mehmet Koca, Veysi Baydar

**Affiliations:** 1 Department of Social Work, Health Science Faculty, Malatya Turgut Özal University, Malatya 44000, Türkiye; mehmet.koca@ozal.edu.tr; 2 Department of Social Work, Faculty of Economics and Administrative Science, Kahramanmaraş Sütçü İmam University, Kahramanmaraş 46100, Türkiye; raufyanardag@ksu.edu.tr; 3 Department of Social Work, Faculty of Economics and Administrative Science, Karabük University, Karabük 78050, Türkiye; veysibaydar@karabuk.edu.tr

**Keywords:** earthquake, posttraumatic growth, psychological resilience, social support

## Abstract

This quantitative study examined the relationships between perceived social support, psychological resilience, and posttraumatic growth (PTG) among university students affected by the 6 February 2023 earthquakes in Türkiye. Utilizing a correlational design, the study tested whether psychological resilience mediated the relationship between perceived social support and PTG. The sample consisted of 769 undergraduate students from Kahramanmaraş Sütçü İmam University and Malatya Turgut Özal University, selected through convenience sampling. Data were collected via standardized instruments: the Multidimensional Scale of Perceived Social Support, the Resilience Scale for Adults, and the Posttraumatic Growth Inventory. A mediation analysis was conducted using the path analysis and bootstrapping methods with the IBM AMOS 24.0 software. The results revealed that perceived social support positively predicted both psychological resilience and PTG, and psychological resilience positively predicted PTG. The mediation analysis confirmed that psychological resilience partially mediated the relationship between perceived social support and PTG. Additionally, significant differences in PTG, resilience, and perceived social support levels were found across gender, housing conditions, psychological impact levels, and access to support. Notably, female students, those who lost loved ones, and those who received psychological or family support reported higher PTG levels. The results emphasize the critical role of social and individual resources in trauma adaptation. It is recommended that post-disaster psychosocial interventions prioritize strengthening both perceived social networks and individual resilience capacities to foster posttraumatic growth in affected populations.

## 1. Introduction

Earthquakes are among the most common natural disasters worldwide. They cause great loss of life and property and have always been a shocking and stressful experience for people due to their unpredictable nature. The 7.7 and 7.6 magnitude earthquakes that occurred in Türkiye on 6 February 2023, with their epicenter in Kahramanmaraş, were recorded as two of the largest and most destructive earthquakes in the country’s history. Official data show that more than 53 thousand people lost their lives, 107 thousand people were injured, hundreds of thousands of buildings were severely damaged or destroyed, and millions of people were left homeless due to these earthquakes (AFAD, 2023).

Large-scale natural disasters such as earthquakes not only cause physical destruction but also leave long-term psychological effects on survivors (Liu et al., 2023). Intense anxiety, fear, sleep problems, helplessness, and depression are among the psychological reactions commonly observed in earthquake victims (Jung & Han, 2023). Studies have shown that individuals who experience trauma after earthquakes may develop psychological problems such as Posttraumatic Stress Disorder (PTSD), depression, and anxiety (Seo & Lee, 2020; Tang et al., 2018). PTSD stands out as the most common psychological problem after earthquakes (Alipour & Ahmadi, 2020). In a meta-analysis study conducted by Dai et al. (2016) examining 46 studies on earthquakes, the incidence of PTSD after an earthquake was found to be 23.66%. The same study revealed that the incidence of PTSD varied depending on variables such as evaluation time, gender, education level, existence of damage to one’s house, loss of loved ones, and presence of physical injury.

From a developmental perspective, university students are in the transition period between late adolescence and adulthood and represent a special group in terms of being affected by traumatic events (Kaya & Bayram, 2024). University students are often overlooked in disaster studies, but they may face unique sources of stress such as being in a developmental stage, academic pressure, and relocation (Kim & Oh, 2018). This population group, which is in the most critical period of their educational life, exhibits different characteristics in terms of coping resources and social support networks. Earthquakes, which can directly affect their family, circle of friends, social environment, and educational environments, can seriously threaten the psychological well-being of these individuals. University students, especially those who continue to live in earthquake zones, face psychological difficulties such as fear, worry, uncertainty, and anxiety about the future. However, despite all these negativities, university students have significant advantages in terms of flexibility and learning capacity thanks to the developmental period they are in. University students, who are in a critical period in terms of physical and mental development, have a high degree of plasticity in terms of cognitive development, which presents a great opportunity for Posttraumatic Growth (PTG) (Xin et al., 2021). This education level of this group and their access to intellectual resources constitute their strengths in the process of understanding and making sense of trauma. Therefore, understanding both risk factors (e.g., anxiety, depressive symptoms) and protective factors (e.g., social support, resilience, education level) for university students after an earthquake is of critical importance for planning interventions for this population.

The concept of PTG is based on the model developed by Tedeschi and Calhoun (1996, 2004). According to this model, traumatic events undermine the individual’s beliefs about life, and after these events, the individual enters a process of re-evaluating their life. By seeking meaning, utilizing social support systems, and activating internal sources of strength, individuals can overcome these challenging times with positive psychological changes. This theoretical framework formed the basis of this study.

Traumatic events often pose a threat to an individual’s psychological well-being. However, not all individuals are affected by such experiences in the same way. Studies pioneered by Tedeschi and Calhoun (1996), from a positive psychology perspective, revealed that some individuals can undergo meaningful and positive psychological changes following traumatic experiences. This process is defined as “PTG” in the literature, where the individual’s coping process with the crisis can include positive transformations in their self-perception, interpersonal relationships, and philosophy of life (Seo & Lee, 2020).

In later studies, PTG was also defined as a mental healing process that occurs through making sense of a traumatic event and developing new hopes for life (Zhou et al., 2015; Xie et al., 2020). Schaefer and Moos (1992) reported three fundamental dimensions of PTG: change in self-perception, change in interpersonal relationships, and change in philosophy of life. Changes in self-perception represent an increased sense of personal strength, resilience, and autonomy, along with the development of new paths and opportunities. Changes in interpersonal relationships, in turn, represent increased compassion, altruism, and closeness in relationships. Finally, changes in philosophy of life encompass potential changes in spiritual and existential beliefs (Taku et al., 2008).

The most widely accepted theoretical model of PTG was developed by Tedeschi and Calhoun (1996, 2004). According to this theoretical model, PTG growth consists of five fundamental dimensions: growth in relationships with others, discovery of new possibilities, personal empowerment, spiritual transformation, and a sense of appreciation for life. Growth in relationships with others involves the individual’s ability to form deeper relationships and a sense of social connectedness through increased empathy. It refers to the discovery of new possibilities and the recognition of new paths and opportunities in life. Personal empowerment refers to an increase in an individual’s belief in their resilience and competence. Spiritual change encompasses a transformation in religious or existential beliefs. Finally, a sense of appreciation for life refers to a greater appreciation of life and small things in life (Jung & Han, 2023; Yoshida et al., 2016; Zoellner & Maercker, 2006). The items on the Posttraumatic Growth Inventory (PTGI), developed by Tedeschi and Calhoun, contain content reflecting these five dimensions. For example, the statement, “I changed my priorities about what is important in life,” refers to the dimension of appreciation for life. Similarly, the item, “I discovered I am stronger than I thought,” reflects the dimension of personal empowerment. Therefore, the theoretical framework adopted in this study not only reflects the multidimensional nature of PTG but also provides a valid basis for interpreting the changes observed after trauma.

In summary, PTG is important in understanding the psychological maturation that occurs in individuals after a process filled with pain, loss, and hardship, and the positive changes observed in various population groups, including university students, after major disasters such as earthquakes (Schulenberg, 2020). In this context, psychological resilience and perceived social support are considered fundamental theoretical elements in understanding the PTG process. Social Cognitive Processing Theory argues that social support plays a critical role in the process of individuals to make sense of traumatic events and cognitive restructuring (Tedeschi & Calhoun, 2004). Similarly, psychological resilience is defined as a key factor determining the capacity of individuals to adapt to challenging life events and is considered directly related to the PTG process (Cann et al., 2010).

Many personal and environmental factors influence the occurrence and degree of PTG. In this context, psychological resilience and perceived social support are considered two critical determinants in the individual’s adaptation process after a traumatic event (Xiao et al., 2016; Alfuqaha et al., 2023).

Although there is no single, agreed-upon functional definition of psychological resilience today, the concept is generally defined as the ability to maintain mental health and adapt positively in the face of adverse living conditions (Herrman et al., 2011). Psychological resilience is a multidimensional concept referring to an individual’s capacity to cope with stressful or traumatic life events. In the literature, this concept is discussed from different perspectives, including process, results, and personal characteristics. When evaluated as a process, psychological resilience is defined as an individual’s adaptive capacity that can change over time and is shaped by environmental factors. According to a results-oriented approach, a person can maintain mental and physical functioning despite negative life experiences. Finally, in the context of a personal characteristic, psychological resilience is defined as the individual’s development and strengthening following difficulties (Chen et al., 2022).

Social support is defined as a concept that expresses the belief of individuals that they are loved, cared for, and valued, as well as their perception that they share mutual obligations in a social network (Gu et al., 2023). Empirical studies have demonstrated the protective effects of social support on PTSD (Neria et al., 2007), burnout (Halbesleben, 2006), depression (Rueger et al., 2016), and subjective well-being (Chu et al., 2010; Wang et al., 2021).

Social support is one of the most emphasized variables among the factors associated with PTG. Social support makes significant contributions to the ability of individuals to reinterpret the events they experience after trauma and structure this process positively (Sztonyk & Formella, 2020). However, findings of studies on social support in the context of PTG are inconsistent due to the diversity of operational definitions. For example, in some studies, this support was defined as perceived support, and the individual’s belief that they can receive help was measured (Uchino, 2009), while in others, the amount of help actually received was evaluated by focusing on the concrete support received (received support) (Helgeson et al., 2000). Additionally, measures based on the size of a social network, such as structural support (network size), were also included in social support assessments (Uchino, 2009). These different definitions may produce different results in studies on the relationship between social support and PTG. Due to this diversity of definitions, while some studies (Dominick et al., 2021; Žukauskienė et al., 2021) found significant and positive relationships between social support and PTG, some other studies (Butler et al., 2005; Kroemeke et al., 2017) could not detect a significant relationship between these two variables. A meta-analysis study conducted by Ning et al. (2023) examining 217 studies on the relationship between social support and PTG demonstrated a positive relationship between social support and PTG in 203 studies. However, the same meta-analysis study also revealed that the relationship between social support and PTG may vary depending on the type of trauma. In the study, it was determined that the relationship between social support and PTG was the weakest in individuals who experienced natural disasters such as earthquakes compared to other types of traumas (illness, grief process, caregiving). This was explained by the likelihood that natural disasters such as earthquakes result in greater physical and financial losses compared to other types of trauma, and post-disaster social support resources often fall short of the needs of surviving individuals (Kaniasty et al., 2020).

Previous studies indicated that variables such as social support and psychological resilience after trauma may be effective mediators on PTG (Ahmadi & Mehrabi, 2020; Finstad et al., 2021). Moreover, previous studies showed a positive relationship between social support and psychological resilience, and psychological resilience was identified as one of the strongest predictors of PTG (Zhang et al., 2017). However, findings regarding the direct effect of social support on PTG are inconsistent in the literature (Finstad et al., 2021). This raises the possibility that the effects of social support on PTG may occur indirectly, for example, through psychological resilience. Therefore, the purpose of this study was to explore the mediating effect of psychological resilience on the relationship between perceived social support and PTG.

In this regard, this study aimed to determine the PTG levels in university students and examine the effects of their perceived social support and psychological resilience levels on their PTG level. In the study, firstly, the correlations between PTG and perceived social support and psychological resilience were examined. Then, the predictive effects of perceived social support and psychological resilience on PTG were analyzed using multiple regression analysis. Moreover, it was aimed to understand the effects of trauma on individuals in more depth by examining the differential effects of some demographic variables such as gender, family loss, and housing status on PTG. The study makes a theoretical contribution to the literature by examining PTG after a natural disaster such as an earthquake in a sample of university students and testing perceived social support and psychological resilience as mediating variables.

The findings of this study, which aims to fill an important gap in understanding the factors affecting PTG among the young adult population after an earthquake, are expected to guide the planning of psychosocial support programs for university students in post-disaster settings.

The hypotheses of this study, which was conducted with university students affected by earthquakes, were as follows:

**H_1_.**  *Perceived social support positively affects PTG.*

**H_2_.**  *Perceived social support positively affects psychological resilience.*

**H_3_.**  *Psychological resilience positively affects PTG.*

**H_4_.**  *Psychological resilience plays a mediating role in the relationship between perceived social support and PTG.*

## 2. Materials and Methods

### 2.1. Design

**Figure 1:**
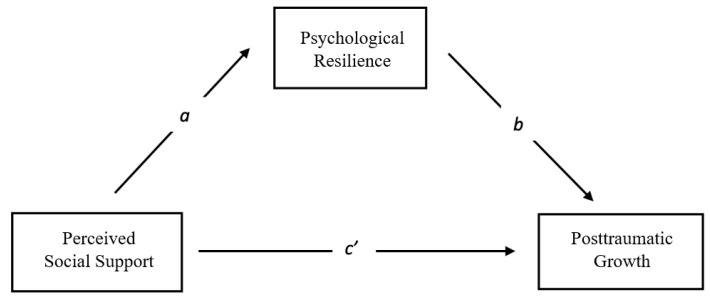
Research model.

This is a quantitative study that aims to examine the relationships between perceived social support, psychological resilience, and PTG. As a quantitative research method, this study adopted a correlational design. To test this model, the mediator variable guideline suggested by Baron and Kenny (1986) was used. The guideline is ultimately based on a basic hypothesis: independent variable (perceived social support) → mediator variable (psychological resilience) → dependent variable (PTG). The study diagram based on the hypotheses is shown in Figure 1.

### 2.2. Sample

The sample consisted of undergraduate students enrolled at Kahramanmaraş Sütçü İmam University and Malatya Turgut Özal University, who had been directly affected by the earthquakes that occurred in Türkiye on 6 February 2023. These institutions were selected based on their proximity to the earthquake zones and the availability of students who had experienced these disasters firsthand.

This study employed a convenience sampling method due to practical considerations such as time limitations, logistical constraints, and ease of access to participants. Rather than drawing from a randomly selected population, participants were recruited based on their availability and willingness to take part in the study, a strategy commonly used in social science research, particularly when working with university students (Stockemer, 2019).

The sample size needed for the study was determined by taking into account the confidence interval (α = 0.05), statistical power (1 − β = 0.80), and effect size (standardized regression coefficients). Calculations for the sample size to be reached were performed using the MedPower Application developed by Kenny (2017) for intermediary models. As a result of the calculations, it was understood that 556 samples needed to be reached according to the indirect effect (α = 0.05; β = 0.06 [standardized indirect effect]; 1 − β = 0.80). Considering these results, it was determined that 769 participants were sufficient for the sample size.

While convenience sampling allowed for efficient data collection, it also introduced certain methodological limitations. Most notably, the lack of randomization reduces the representativeness of the sample, and the findings should therefore be interpreted with caution and not generalized beyond the sample (Andrade, 2020).

#### 2.2.1. Inclusion Criteria

Participants were eligible for inclusion in the study if they were 18 years of age or older, currently enrolled in an undergraduate program at either of the two target universities, had been directly affected by the earthquakes that occurred on 6 February 2023, and provided informed consent to voluntarily participate in the study.

#### 2.2.2. Exclusion Criteria

Prior to participation, individuals were asked whether they had ever been diagnosed with a serious psychiatric disorder or were currently undergoing psychiatric treatment. Those who disclosed such conditions were excluded from the study to adhere to ethical standards and minimize the risk of psychological distress during the data collection process.

### 2.3. Data Collection Instruments

The data collection instruments consisted of four sections: (1) a demographic information form, (2) the Multidimensional Scale of Perceived Social Support, (3) the Resilience Scale for Adults, and (4) the Posttraumatic Growth Inventory.

1. Demographic Information Form: This form consisted of 15 questions. Some of these questions aimed to identify the participants based on variables such as gender, age, family income, and place of residence, while others facilitated an understanding of the earthquake’s impact on them. Such questions included the psychological effects of the earthquake, the availability of psychological support, and the factors facilitating coping.
2. Multidimensional Scale of Perceived Social Support (MSPSS): The scale was developed by Zimet et al. (1988) and adapted into a revised Turkish form by Eker et al. (2001). The scale focuses on social support received from family, friends, and significant others and consists of 12 items. As scores on the scale increase, perceived social support also increases. In the study by Eker et al. (2001), the Cronbach’s alpha internal consistency coefficient for the entire sample of the scale defined as a three dimensional construct was found to be 0.85 for the family dimension, 0.88 for the friends dimension, and 0.92 for the significant others dimension. The reliability coefficient of the overall scale was 0.89. In this study, the Cronbach’s alpha coefficient of the overall scale was obtained as 0.90.
3. Resilience Scale for Adults (RSA): The scale was developed by Friborg et al. (2003) and adapted to Turkish by Basım and Çetin (2011). This scale consists of 33 items and measures psychological resilience in six dimensions. These dimensions are personal competence, future perception, personal structure, social competence, family cohesion, and social support. The scale was applied to two samples: students and employees. According to the analyses, Cronbach’s alpha coefficients for the dimensions ranged from 0.66 to 0.81 in the student group and from 0.68 to 0.79 in the employee group. For the overall scale, this coefficient was found to be 0.86 in both the student and employee groups (Basım & Çetin, 2011). In this study, psychological resilience was evaluated in a unidimensional manner, and the Cronbach’s alpha coefficient was found to be 0.83. The scale is scored in such a way that higher scores indicate greater psychological resilience.
4. Posttraumatic Growth Inventory (PTGI): This inventory was developed by Tedeschi and Calhoun (1996). The psychometric properties of the Turkish version were examined by Kağan et al. (2012). The inventory, consisting of 21 items, aims to measure positive changes and developments after trauma. To examine its psychometric properties, the inventory was administered to high school and university students. The inventory consists of the dimensions of change in self-perception (α = 0.88), change in philosophy of life (α = 0.78), and change in relationships (α = 0.77). The Cronbach’s alpha coefficient obtained for the overall construct of the scale was reported as 0.92. In this study, the Cronbach’s alpha coefficient for the overall scale was found to be 0.90. As scores on the scale increase, PTG also increases.

### 2.4. Procedure

Necessary legal and ethical permissions were obtained for the study. In this regard, the study was carried out with the approval of Malatya Turgut Özal University Social Sciences, Humanities, and Administrative Sciences Ethics Committee. The research process was designed and implemented in accordance with the ethical principles of the Declaration of Helsinki. The data were collected through questionnaires administered to students studying at Malatya Turgut Özal University (MTU) and Kahramanmaraş Sütçü İmam University (KSU) campuses in the provinces affected by the earthquakes of 6 February 2023, in classroom environments between 1 April and 15 May 2025, after obtaining their informed consent.

### 2.5. Data Analysis

The IBM SPSS 26 and IBM AMOS 24 package programs were used for the statistical analyses of the data. Necessary investigations were carried out to provide the prerequisites for testing the research model. Cronbach’s alpha coefficient was considered a criterion for the reliability of the scales, and the Pearson Product-Moment Correlation Coefficient was used to determine the linear relationships between the variables before path analysis (Pallant, 2020). For the mediation analysis, path analysis was conducted based on the observed variables. The mediation model was estimated using the Maximum Likelihood (ML) method (Kline, 2016). Confidence intervals were taken into account for the significance of the effects, and the model was tested using the 5000 bootstrap resampling method within a 95% confidence interval. In this method, the absence of a ‘0’ value within the lower and upper limits of the confidence interval indicates a mediation effect (Preacher & Hayes, 2008).

Additionally, differences in the variables analyzed in the study across demographic characteristics were examined using independent-samples *t*-tests and one-way analysis of variance (ANOVA), as appropriate. When the ANOVA indicated statistically significant group differences, post hoc comparisons were conducted using Bonferroni correction to identify specific group differences.

## 3. Results

A significant portion of the participants were women (n = 245, 70.6%) and young people (*M_age_* = 21, *SD* = 2.66). Most were first or second year students, with approximately equal representation from Kahramanmaraş Sütçü İmam University and Malatya Turgut Özal University. The participants primarily lived in dormitories and reported that their parents were living together. Reported family income levels were generally close to the national minimum wage. Most participants had three or more siblings. Further demographic details of the participants are provided in Table 1.

While 79.1% of the participants stated that they lived in dormitories, 16% lived with their families. Additionally, 85.7% of the participants stated that their parents were still married and living together, 6.6% stated that their parents were divorced, 4.7% stated that their fathers had passed away, and 2.2% stated that their mothers had passed away. As seen here, the majority of the participants lived in dormitories, and the majority had parents who were living together. The majority of the participants had three siblings (29.6%). Furthermore, the proportions of those with four siblings and those with five or more siblings were almost the same, at approximately 24% each (Table 1).

A significant portion of the participants (38.9%) reported being deeply affected by the earthquakes in a psychological sense. Despite this, the vast majority (88.7%) stated that they had not received any form of psychological support. Additionally, nearly one in three participants (32.1%) experienced the loss of a family member or close relative. While only 3.5% reported that their homes were completely destroyed, more than half indicated varying degrees of structural damage. Most participants (83.4%) remained in their original place of residence following the disaster. As for coping strategies, 32.5% reported receiving support from family members, while 26.5% turned to spirituality. Further details are presented in Table 2.

### 3.1. Differences in Examined Variables Based on Sociodemographic and Earthquake-Related Characteristics

Significant differences were observed in the perceived social support levels of the participants based on their gender (*t* = 5.473; *p* < 0.05), number of siblings (*F* = 2.903; *p* < 0.05), status of having damage to their house (*F* = 5.605; *p* < 0.001), trauma history (*t* = −2.871; *p* < 0.05), psychological support status (*t* = −2.833; *p* < 0.01), and coping methods (*F* = 6.799; *p* < 0.001). In this sense, the female participants had higher scores in comparison to the male participants. The participants with two siblings had a higher perception of social support than those with five or more siblings. There were significant differences in perceived social support levels between participants with slightly damaged or undamaged houses and those with severely damaged houses. In particular, those with homes having minor or no damage reported higher perceived social support. Those with a trauma history reported lower perceived social support than those without, while those who unexpectedly received psychological support reported lower perceived social support than those who did not. The participants who received support from their relatives had higher perceived social support levels. Receiving support from relatives differed significantly from receiving professional support, doing volunteer work, and working with NGOs. Moreover, although it did not show a significant difference among the coping elements compared to the other groups, spirituality was seen as an important element (Appendix A, Table A1).

Perceived social support did not show significant differences according to the characteristics of the participants such as their place of residence (*F* = 1.802; *p* > 0.05), parental status (*F* = 1.494; *p* > 0.05), psychological impact status (*F* = 2.541; *p* > 0.05), relocation status (*t* = 0.157; *p* > 0.05), or status of losing a loved one (*t* = 1.017; *p* > 0.05) (Appendix A, Table A1).

The psychological resilience levels of the participants displayed significant differences based on gender (*t* = 6.895; *p* < 0.001), parental status (*F* = 4.502; *p* < 0.001), number of siblings (*F* = 6.031; *p* < 0.001), level of psychological impact (*F* = 3.675; *p* < 0.05), status of damage to one’s house (*F* = 7.916; *p* < 0.001), trauma history (*t* = −3.743; *p* < 0.001), psychological support (*t* = −4.487; *p* < 0.001), and coping methods (*F* = 4.607; *p* < 0.001). In group-level differences, women had higher psychological resilience than men. Those whose families lived together exhibited higher psychological resilience than those whose parents were divorced. Those with one sibling showed lower psychological resilience than other groups. Those with two siblings had the highest psychological resilience scores. While those who stated that they were deeply psychologically affected by the earthquakes had higher scores, this group differed from those less affected. The participants whose houses were destroyed had lower psychological resilience levels than those in other categories. While the participants without a history of trauma reported higher resilience than those with one, those who reported not receiving psychological support reported higher resilience than those who did. The participants who received support from relatives had higher scores among the categories established based on coping methods, and this group showed a significant difference compared to the group of participants who took part in voluntary work (Appendix A, Table A2).

No significant differences were found in the psychological resilience levels of that participants based on characteristics such as their place of residence (*F* = 1.268; *p* > 0.05), relocation status (*F* = −0.761; *p* > 0.05), and status of losing a loved one (*F* = 1.469; *p* > 0.05) (Appendix A, Table A2).

The PTG levels of the participants differed significantly according to some demographic variables of theirs. These variables were gender (*t* = 3.701; *p* < 0.001), place of residence (*F* = 3.981; *p* < 0.01), level of psychological impact (*F* = 9.719; *p* < 0.001), status of losing a loved one (*t* = 2.031; *p* < 0.05), psychological support (*t* = 2.490; *p* < 0.01), and coping elements (*F* = 3.758; *p* < 0.01). In the comparisons among the groups, women showed higher PTG levels than men. The participants who were staying with friends exhibited higher PTG compared to those who lived in dormitories, the participants who were affected more by the earthquakes exhibited higher PTG compared to those who were less affected, those who lost a loved one exhibited higher PTG compared to those who did not, those who received psychological support exhibited higher PTG compared to those who did not, and those who received support from relatives exhibited higher PTG compared to those who did not. However, no differences were found according to variables such as parental status (*F* = 0.553; *p* > 0.05), number of siblings (*F* = 1.968; *p* > 0.05), status of having damage to one’s house (*F* = 0.533; *p* > 0.05), relocation status (*F* = −0.715; *p* > 0.05), or trauma history (*F* = −0.610; *p* > 0.05) (Appendix A, Table A3).

### 3.2. Relationships Between Variables

Table 3 presents the results of the zero-order correlation analysis. In the correlation analysis, a moderately (Cohen, 1988) significant positive relationship was found between perceived social support and psychological resilience (*r* = 0.49; *p* < 0.01). Likewise, a moderately significant positive relationship was found between perceived social support and PTG (*r* = 0.38; *p* < 0.01). A weak and positive relationship was found between psychological resilience and PTG (*r* = 0.28; *p* < 0.01). These results indicated that there were significant and positive relationships between all variables (Table 3).

The effect of perceived social support on PTG (total effect) was statistically significant (*β* = 0.38; *p* < 0.001; CI [0.31–0.45]). After psychological resilience was included in the model, this effect decreased (direct effect), but statistical significance remained (*β* = 0.32; *p* < 0.001; CI [0.23–0.40]). The effect of psychological resilience on PTG was also found to be significant (*β* = 0.13; *p* < 0.001; CI [0.04–0.21]).

**Figure 2:**
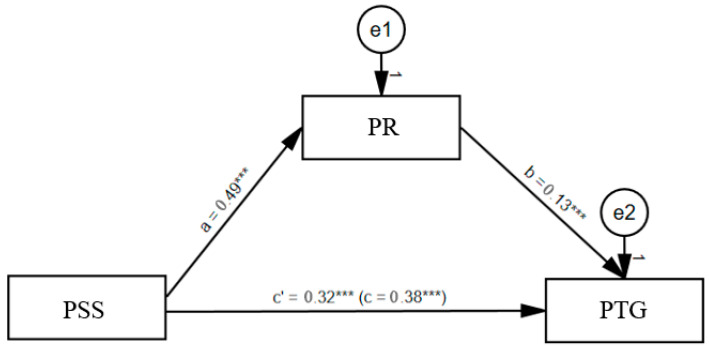
Standardized regression coefficients in the mediation model. Note. PSS: Perceived Social Support; PR: Psychological Resilience; PTG: Posttraumatic Growth, *** *p* < 0.001.

As a result of the analyses, it was determined that the indirect effect representing the mediation effect was significant (standardized indirect effect = 0.06; CI [0.02–0.10]). These results indicated that psychological resilience mediated the relationship between perceived social support and PTG. The model as a whole explained 16% of the total variance in the variable in question (*F* = 70.35; *p* < 0.001). The standardized coefficients for the model are summarized in Figure 2, and the unstandardized coefficients are summarized in Table 4.

As a result of the analyses, it was observed that perceived social support positively affected PTG and psychological resilience, and psychological resilience positively affected PTG. Moreover, it was confirmed that psychological resilience played a mediating role in the relationship between perceived social support and PTG. With these results, all hypotheses formed in the study, expressed as H_1_, H_2_, H_3_, and H_4_, were supported.

## 4. Discussion

Earthquakes are natural disasters that cause both great loss of life and psychological traumatic effects on individuals (Karal et al., 2024). Identifying these psychological traumas is not as easy as identifying deaths and financial losses caused by disasters. The detection of psychological effects, which are relatively more subtle, is important for individuals to adapt to social life and determine what needs to be done. This study examined the relationships between perceived social support, psychological resilience, and PTG in university students after major earthquakes using a quantitative design. The results revealed that levels of PTG, psychological resilience, and perceived social support significantly differed across certain demographic variables.

The timing of data collection is a crucial factor in PTG research, as growth processes often evolve over time. In this study, data were collected approximately 26 months after the aforementioned earthquakes. Previous studies suggested that PTG tended to increase as more time elapsed post-trauma, allowing for reflection and cognitive processing (Taku et al., 2008; Helgeson et al., 2006). Therefore, the results should be interpreted with the understanding that PTG levels observed in this sample may have been influenced by the time that elapsed since the disaster.

The results of this study showed that the female participants reported significantly higher levels of perceived social support, psychological resilience, and PTG compared to their male counterparts. This difference may be attributed to the greater tendency of women to maintain social relationships and express their emotional responses to traumatic experiences more openly (Tamres et al., 2002). The findings indicated that the participants with two siblings exhibited higher levels of perceived social support and psychological resilience. This outcome aligned with previous studies suggesting that having a larger number of siblings may lead to competition over limited family resources, whereas having too few siblings may restrict opportunities for social interaction and support (Furman & Buhrmester, 1985).

In this study, it was determined that 32.1% of the participants experienced loss of family and loved ones due to the earthquakes, and 61% of the participants suffered varying degrees of damage to their houses, ranging from minor damage to complete collapse during the earthquakes. The rate of participants psychologically affected by the earthquakes was 92.3%. The finding that the rate of psychological impact was higher than the rate of loss of life and property supported the conclusion that psychologically traumatic events affected not only those living in the earthquake zone and experiencing the earthquakes but also those who were exposed to news from the earthquake zone (Ciller et al., 2023).

Despite the high rate of those psychologically affected by the earthquakes, it was determined that only 3.5% of the participants received professional support, while 32.5% received support from their relatives to cope with this traumatic event. A significant relationship was found between support received from close social networks and levels of PTG, indicating that such support contributes positively to the enhancement of PTG. This finding may be interpreted as evidence that the sense of social connectedness following trauma plays a beneficial role in the recovery process.

After Hurricane Katrina, perceived social support was determined to be a protective factor against depression and generalized anxiety disorder five years after the traumatic event (Cherry et al., 2015). It was stated that social support is an effective way to alleviate psychological disorders and cope with insomnia and stress reactions (Yao et al., 2024; Shuwiekh et al., 2018). In a study conducted with Chinese university students during the COVID-19 pandemic, it was determined that the students who received support from their families and peers overcame the trauma caused by the outbreak more easily and had better learning and academic performance at home (Raaper et al., 2021). In another study conducted after the 2023 Kahramanmaraş earthquakes in Türkiye, it was determined that individuals affected by the earthquakes needed social support to find meaning in life and feel hopeful about their situation (Yalnızca-Yıldırım, 2024).

Although these results showed that social support was important for increasing the ability of individuals to cope with traumatic events and combat stress, it was determined that only 38% of the participants in this study received support from professionals or relatives. There is a need to implement widespread post-disaster support programs to increase the level of coping with post-traumatic stress and psychological resilience. These programs should be included in disaster preparedness plans that cover the entire society, and it is important to begin implementing these programs quickly after a disaster.

In the study, a positive and moderately significant relationship was found between perceived social support and PTG (*r* = 0.38; *p* < 0.01). A meta-analysis study also showed a moderate correlation between social support and PTG with a mean effect size of *r* = 0.26 (Prati & Pietrantoni, 2009). The results of these studies were similar, and it can be stated that increasing the social support individuals receive from their family, friends, and other social circles helps them perceive traumatic events as less stressful and derive a positive meaning from their traumatic experiences (Aliche et al., 2019).

Psychological resilience and PTG are two important psychological outcomes of positive responses to trauma, and the relationship between them is still controversial (Li et al., 2025). There are studies showing that PTG levels are higher in individuals with high levels of psychological resilience (Lee et al., 2019; Wu et al., 2015), as well as studies suggesting that resilience may be negatively correlated with PTG (Long et al., 2020). It was reported that people with high resilience may exhibit less PTG in the face of trauma because they can cope with difficulties (Tedeschi & Calhoun, 2004). In this study, a weak and positive relationship was found between psychological resilience and post-traumatic growth (*r* = 0.28; *p* < 0.01). Xu et al. (2024) found that the psychological resilience of university students during the COVID-19 pandemic had a significant and positive correlation with their PTG (*r*  =  0.414). A 10-year cohort study conducted after the Wenchuan earthquake reported that individuals in the high resilience group were more likely to report higher future PTG compared to the low resilience group, especially among males (Chen et al., 2022). Mesidor and Sly (2019) found a significant positive correlation between resilience and PTG in their study after the earthquakes that occurred in Haiti in 2010. It is possible that resilient individuals used unique strategies to cope with the destruction left by the earthquake, which made them feel stronger and thus encouraged psychological growth.

Kanbay et al. (2025) found a weak positive relationship (*r* = 0.229) between social support and psychological resilience in their study after the 6 February earthquakes in Türkiye. Xi et al. (2020) reported a positive correlation (*r* = 0.36) between resilience and social support in their study after the Jiuzhaigou earthquake. In this study, a moderate, positive, and significant relationship was obtained between perceived social support and psychological resilience (*r* = 0.49). The results of different studies were similar in terms of the direction of the correlations reported, and it can be argued that social support and psychological resilience affected each other.

According to the analysis results, social support positively affected PTG and psychological resilience. It was also found that psychological resilience played a mediating role in the relationship between perceived social support and PTG. It was concluded that perceived social support and PTG were partly affected by psychological resilience. It can be interpreted from here that social support partially reduces the individual’s anxiety levels in coping with difficulties, and as a result, the individual’s reduced stress levels increases their ability to cope with problems and fighting power, and psychological resilience partially affects this interaction.

The study had limitations arising from its cross-sectional design and sampling method. These results are limited to the population of the study. It is recommended to carry out longitudinal cohort studies to determine the effects of traumatic events experienced by individuals and how social support, psychological resilience, and PTG change over time.

## 5. Conclusions

People are affected by earthquakes at different degrees. This study examined perceived social support, psychological resilience, and posttraumatic growth (PTG) among university students affected by the Kahramanmaraş-centered earthquakes, yielding significant findings. It was determined that positive psychological change following trauma is influenced not only by individual factors but also by social and contextual dynamics. In particular, perceived social support was found to have a positive effect on both psychological resilience and PTG, while psychological resilience also positively influenced PTG. Furthermore, psychological resilience was confirmed to serve a mediating role in the relationship between perceived social support and PTG. According to the results, female participants, those living with friends, those who experienced the loss of a loved one, and those who received psychological support reported higher levels of PTG. Additionally, support received from close social circles and the use of spiritual coping strategies emerged as significant contributors to PTG. These findings underscored that the responses of individuals to trauma vary significantly, and such differences must be acknowledged in post-disaster recovery processes. In this regard, it is recommended that public institutions and civil society organizations should maintain a continuous commitment to building effective social support networks before, during, and after disasters, while taking individual differences into account. Moreover, it is recommended that feasible and actionable plans be developed to provide immediate social support in the aftermath of disasters.

## Figures and Tables

**Table 1:** Demographic characteristics of the participants.

Variables		F (n = 769)	%
**Gender**	Female	543	70.6
	Male	226	29.4
**University**	KSU	390	50.7
	MTU	379	49.3
**Class**	1st year	246	32
	2nd year	235	30.6
	3rd year	190	24.7
	4th year	98	12.7
**Family Income**	Under 10 thousand TL	57	7.4
	10–20 thousand TL	157	20.4
	20–30 thousand TL	190	24.7
	30–40 thousand TL	137	17.8
	40–60 thousand TL	160	20.8
	60 thousand TL and above	68	8.8
**Living**	In student dormitory	608	79.1
	With family	124	16.1
	With friend(s)	16	2.1
	Other	21	2.7
**Parental Status**	Together	659	85.7
	Divorced	51	6.6
	Mother deceased	17	2.2
	Father deceased	36	4.7
	Both are deceased	6	0.8
**Number of Siblings**	1	36	4.7
	2	131	17
	3	228	29.6
	4	185	24.1
	5 or above	189	24.6
	**Mean**	**SD**	**range**
**Age**	21.15	2.66	28

Note. TL: Turkish Lira.

**Table 2:** Earthquake-related characteristics of the participants.

Variables		F (n = 769)	%
**Psychological Impact**	Not affected at all	59	7.7
	Slightly affected	256	33.3
	Highly affected	299	38.9
	Extremely affected	155	20.2
**Condition of House**	It collapsed during the earthquake	27	3.5
	Heavily damaged	104	13.5
	Moderately damaged	86	11.2
	Slightly damaged	252	32.8
	Undamaged	300	39
**Location Change Status**	Yes	128	16.6
	No	641	83.4
**Loss of a loved one**	Yes	247	32.1
	No	522	67.9
**Trauma History**	Yes	315	41
	No	454	59
**Psychological Support**	Yes	87	11.3
	No	682	88.7
**Coping Elements**	Got professional support	27	3.5
	Participated in aid efforts as a volunteer	71	9.2
	Participated in aid efforts with NGOs	16	2.1
	Received support from loved ones	250	32.5
	Spirituality	204	26.5
	Other	201	26.1

**Table 3:** Mean, standard deviation and zero-order correlations of variables.

Variables	*M*	*SD*	1	2	3
1. Perceived Social Support	4.86	1.38	-		
2. Psychological Resilience	3.56	0.57	0.49 **	-	
3. Posttraumatic Growth	2.83	0.87	0.38 **	0.28 **	-

** *p* < 0.01.

**Table 4:** Mediation model analysis results.

Predictor Variables	Outcome Variables			
	Psychological Resilience		Post-Traumatic Growth	
	*B*	*SE*	*B*	*SE*
Perceived Social Support (path a)	0.20 ***	0.01	-	-
Psychological Resilience (path b)	-	-	0.19 ***	0.06
Perceived Social Support (path c′)	-	-	0.20 ***	0.02
Perceived Social Support (path c)	-	-	0.24 ***	0.03
Indirect Effect (PSS→PR→PTG)	-	-	0.04 (0.01–0.06)	
*R* ^2^	0.24		0.16	

Note. *** *p* < 0.001. *B*: Unstandardized beta coefficients, *SE*: Standard Error, values in parentheses are lower and upper confidence interval values (95%).

## Data Availability

The data are kept confidential. Data are available from the corresponding author upon reasonable request.

## Abbreviations

The following abbreviations are used in this manuscript:

MTU	Malatya Turgut Özal University
KSU	Kahramanmaraş Sütçü İmam University
PSS	Perceived Social Support
PR	Psychological Resilience
PTG	Posttraumatic Growth
PTGI	Posttraumatic Growth Inventory
PTSD	Posttraumatic Stress Disorder
TL	Turkish Lira

## Appendix A

**Table A1:** Independent-samples *t*-test and one-way ANOVA results for differences in perceived social support across demographic and earthquake-related variables.

Variables	Gender										*F/t*
**PSS**	**Male**				**Female**						
	*M*		*SD*		*M*			*SD*			
	4.44 ^2^		1.23		5.03 ^1^			1.40			5.473 *
	**Place of Stay**										
	**Dormitory**		**Family**		**Friend**		**Other**				
	*M*	*SD*	*M*	*SD*	*M*	*SD*	*M*		*SD*		
	4.80	1.37	4.99	1.34	5.01	1.18	5.4		1.89		1.802
	**Parental Status**										
	**Together**		**Divorced**		**Mother passed away**		**Father passed away**		**Parents passed away**		
	*M*	*SD*	*M*	*SD*	*M*	*SD*	*M*	*SD*	*M*	*SD*	
	4.88	1.42	4.66	1.27	4.53	1.06	4.96	1.02	3.80	1.09	1.494
	**Number of Siblings**										
	1		2		3		4		5≥		
	*M*	*SD*	*M*	*SD*	*M*	*SD*	*M*	*SD*	*M*	*SD*	
	4.72	1.61	5.09 ^1^	1.41	4.92	1.33	4.92	1.32	4.59 ^2^	1.43	2.903 *
	**Psychological Impact**										
	**I was not affected at all**				**I was slightly affected**		**I was deeply affected**				
	*M*		*SD*		*M*	*SD*	*M*		*SD*		
	4.73		1.71		4.72	1.38	4.95		1.33		2.541
	**Damage to the House**										
	**Slight Damage/No Damage**		**Moderate Damage**		**Severe Damage**				**Destroyed**		
	*M*	*SD*	*M*	*SD*	*M*		*SD*		*M*	*SD*	
	4.97 ^1^	1.38	4.71	1.21	4.43 ^2^		1.42		4.52	1.39	5.605 ***
	**Relocation**										
	Yes				No						
	*M*		*SD*		*M*		*SD*				
	4.88		1.35		4.86		1.39				0.157
	**Loss of a loved one**										
	Yes				No						
	*M*		*SD*		*M*		*SD*				
	4.93		1.37		4.82		1.39				1.017
	**Trauma History**										
	Yes				No						
	*M*		*SD*		*M*		*SD*				
	4.69 ^2^		1.41		4.98 ^1^		1.35				−2.871 *
	**Psychological Support**										
	Yes					No					
	*M*		*SD*		*M*		*SD*				
	4.46 ^2^		1.30		4.91 ^1^		1.39				−2.833 **
	**Coping Elements**										
	Professional support		Volunteer work		Working with NGOs		Support from relatives		Spirituality		
	*M*	*SD*	*M*	*SD*	*M*	*SD*	*M*	*SD*	*M*	*SD*	
	4.25 ^2^	1.48	4.60 ^2^	1.31	4.18 ^2^	0.99	5.18 ^1^	1.24	4.99	1.31	6.799 ***

Note. PSS: Perceived Social Support, * *p* < 0.05, ** *p* < 0.01, *** *p* < 0.001, Bonferroni α = 0.05; ^1 > 2^.

**Table A2:** Independent-samples *t*-test and one-way ANOVA results for differences in psychological resilience across demographic and earthquake-related variables.

Variables	Gender										*F/t*
**PR**	**Male**				**Female**						
	*M*		*SD*		*M*				*SD*		
	3.34 ^2^		0.54		3.65 ^1^				0.56		6.895 ***
	**Place of Stay**										
	**Dormitory**		**Family**		**Friend**				**Other**		
	*M*	*SD*	*M*	*SD*	*M*		*SD*		*M*	*SD*	
	3.56	0.56	3.54	0.59	3.37		0.40		3.73	0.73	1.268
	**Parental Status**										
	**Together**		**Divorced**		**Mother passed away**		**Father passed away**		**Parents passed away**		
	*M*	*SD*	*M*	*SD*	*M*	*SD*	*M*	*SD*	*M*	*SD*	
	3.59 ^1^	0.57	3.34 ^2^	0.61	3.26	0.42	3.39	0.57	3.43	0.58	4.502 ***
	**Number of Siblings**										
	1		2		3		4		5≥		
	*M*	*SD*	*M*	*SD*	*M*	*SD*	*M*	*SD*	*M*	*SD*	
	3.19 ^2^	0.46	3.67 ^1^	0.62	3.56 ^1^	0.59	3.61 ^1^	0.54	3.50 ^1^	0.53	6.031 ***
	**Psychological Impact**										
	**I was not affected at all**				**I was slightly affected**		**I was deeply affected**				
	*M*		*SD*		*M*	*SD*	*M*		*SD*		
	3.49		0.68		3.49 ^2^	0.52	3.61 ^1^		0.58		3.675 *
	**Damage to the House**										
	**Slight Damage/No Damage**		**Moderate Damage**		**Severe Damage**				**Destroyed**		
	*M*	*SD*	*M*	*SD*	*M*		*SD*		*M*	*SD*	
	3.62 ^1^	0.57	3.39 ^1^	0.54	3.43 ^1^		0.53		3.32 ^2^	0.66	7.916 ***
	**Relocation**										
	Yes				No						
	*M*		*SD*		*M*				*SD*		
	3.53		0.59		3.57				0.57		−0.761
	**Loss of a loved one**										
	Yes				No						
	*M*		*SD*		*M*				*SD*		
	3.60		0.59		3.54				0.56		1.469
	**Trauma History**										
	Yes				No						
	*M*		*SD*		*M*				*SD*		
	3.47 ^2^		0.56		3.62 ^1^				0.57		−3.743 ***
	**Psychological Support**										
	Yes				No						
	*M*		*SD*		*M*				*SD*		
	3.34 ^2^		0.46		3.59 ^1^				0.58		−4.487 ***
	**Coping Elements**										
	Professional support		Volunteer work		Working with NGOs		Support from relatives		Spirituality		
	*M*	*SD*	*M*	*SD*	*M*	*SD*	*M*	*SD*	*M*	*SD*	
	3.38	0.65	3.45 ^2^	0.58	3.38	0.39	3.69 ^1^	0.52	3.61	0.59	4.607 ***

Note. PR: Psychological Resilience, * *p* < 0.05, *** *p* < 0.001, Bonferroni α = 0.05; ^1 > 2^.

**Table A3:** Independent-samples *t*-test and one-way ANOVA results for differences in post-traumatic growth across demographic and earthquake-related variables.

Variables	Gender										*F/t*
**PTG**	**Male**				**Female**						
	*M*		*SD*		*M*		*SD*				
	2.65 ^2^		0.93		2.91 ^1^		0.83				3.701 ***
	**Place of Stay**										
	**Dormitory**		**Family**		**Friend**		**Other**				
	*M*	*SD*	*M*	*SD*	*M*	*SD*	*M*		*SD*		
	2.78 ^2^	0.86	2.99	0.80	3.34 ^1^	0.73	2.93		1.32		3.981 **
	**Parental Status**										
	**Together**		**Divorced**		**Mother passed away**		**Father passed away**		**Parents passed away**		
	*M*	*SD*	*M*	*SD*	*M*	*SD*	*M*	*SD*	*M*	*SD*	
	2.84	0.88	2.82	0.75	2.56	0.66	2.94	0.99	2.78	0.62	0.553
	**Number of Siblings**										
	1		2		3		4		5≥		
	*M*	*SD*	*M*	*SD*	*M*	*SD*	*M*	*SD*	*M*	*SD*	
	2.78	0.97	2.86	0.87	2.92	0.81	2.87	0.81	2.69	0.95	1.968
	**Psychological Impact**										
	**I was not affected at all**				**I was slightly affected**		**I was deeply affected**				
	*M*		*SD*		*M*	*SD*	*M*		*SD*		
	2.74		1.27		2.65 ^2^	0.85	2.94 ^1^		0.79		9.719 ***
	**Damage to the House**										
	**Slight Damage/No Damage**		**Moderate Damage**		**Severe Damage**				**Was Destroyed**		
	*M*	*SD*	*M*	*SD*	*M*		*SD*		*M*	*SD*	
	2.84	0.90	2.90	0.72	2.77		0.78		2.72	0.93	0.533
	**Relocation**										
	Yes				No						
	*M*		*SD*		*M*			*SD*			
	2.78		0.89		2.84			0.86			−0.715
	**Loss of a loved one**										
	Yes				No						
	*M*		*SD*		*M*			*SD*			
	2.93 ^1^		0.81		2.79 ^2^			0.89			2.031 *
	**Trauma History**										
	Yes				No						
	*M*		*SD*		*M*			*SD*			
	2.81		0.88		2.85			0.86			−0.610
	**Psychological Support**										
	Yes				No						
	*M*		*SD*		*M*			*SD*			
	3.05 ^1^		0.79		2.81 ^2^			0.87			2.490 **
	**Coping Elements**										
	Professional support		Volunteer work		Working with NGOs		Support from relatives		Spirituality		
	*M*	*SD*	*M*	*SD*	*M*	*SD*	*M*	*SD*	*M*	*SD*	
	3.21	0.69	2.96	0.71	3.03	0.69	3.05 ^1^	0.70	2.79 ^2^	0.89	3.758 **

Note. PTG: Posttraumatic Growth, * *p* < 0.05, ** *p* < 0.01, *** *p* < 0.001, Bonferroni α = 0.05; ^1 > 2^.
